# #BodyPositivity: The Role of Body Appreciation and Body Mass Index for Muscle Dysmorphia, Body Attitudes and Exercise Motives Among Men

**DOI:** 10.3390/nu17071177

**Published:** 2025-03-28

**Authors:** Kamila Czepczor-Bernat, Adriana Modrzejewska, Justyna Modrzejewska

**Affiliations:** 1 Department of Pediatrics, Pediatric Obesity and Metabolic Bone Diseases, Faculty of Medical Sciences in Katowice, Medical University of Silesia, 40-055 Katowice, Poland; 2 Department of Medical Anthropology, Faculty of Medical Sciences in Katowice, Medical University of Silesia, 40-055 Katowice, Poland; adriana.modrzejewska@sum.edu.pl; 3 Institute of Pedagogy, University of Bielsko-Biała, 43-300 Bielsko-Biala, Poland; jmodrzejewska@ubb.edu.pl

**Keywords:** body appreciation, body attitudes, body mass index, body positivity, exercise motives, men, muscle dysmorphia

## Abstract

**Objectives**: The main aim of this study was to investigate the hypothesis that men with high body appreciation and healthy (normal) weight would have significantly lower levels of muscle dysmorphia and the non-adaptive (negative) multiple dimensions of body attitudes, as well as higher levels of the selected pro-health and adaptive exercise motives, than those men with low body appreciation and excess body weight. **Methods**: A total of 374 Polish men (*M*_age_ = 28.96 ± 8.52) completed the following questionnaires: (a) the Body Appreciation Scale-2, (b) the Muscle Dysmorphic Disorder Inventory, (c) the Male Body Attitude Scale, and (d) the Exercise Motivations Inventory-2. **Results**: By analyzing the most important findings, it was discovered, as hypothesized, that significant differences (in terms of most of the assessed scale/subscales) were found between men who had high body appreciation and healthy weight (Cluster 4), and men with low body appreciation and excess body weight (Cluster 3). Cluster 4 (vs. Cluster 3) was characterized as follows: (a) considering muscle dysmorphia, these participants had lower levels in terms of the total score and subscale of appearance intolerance; (b) they had fewer non-adaptive (negative) body attitudes, with lower total scores and lower scores on two subscales (body fat and height); (c) for the selected pro-health and adaptive exercise motives, Cluster 4 had higher levels on all subscales. **Conclusions**: Our results show that holding views in line with positive body image is also beneficial for the functioning of adult men. However, further research needs to be conducted in this area to determine whether the content in the interventions and prevention activities for both sexes should be the same and what factors should be taken into account in order to influence excessive fixation on a muscularity.

## 1. Introduction

Body positivity (most often marked on social media as #BodyPositive/#BoPo) is a social movement focusing on the topic of body acceptance, regardless of differences between people in this context (concerning, among others, body size and shape, skin tone, gender and physical abilities) [1,2,3]. It is a social movement that challenges the modern pressure to achieve commonly promoted “standards” of beauty. The main ideas of this movement focus on appreciating the functionality and health of the human body, instead of focusing excessively on physical appearance [1,2,3]. Research shows that these attitudes may be associated with healthier eating behaviors and a more adaptive attitude to one’s own body [1,4,5]. Content related to #BodyPositivity also refers to taking care of the body through physical activity, which is based on the assumption that exercise is a form of taking care of physical and mental health, and the chosen type of activity is a source of pleasure [6,7,8,9].

A questionnaire that seems to reflect well some selected aspects of the attitudes related to #BodyPostivity is the Body Appreciation Scale 2 [10]. This measure is related to the attitude towards the body—body appreciation—that we wanted to measure. After reviewing the literature, we identified that this measure more strongly reflects certain selected views related to #BodyPostivity; it should be emphasized, however, that it does not directly measure all views related to the mentioned social movement, and the concepts of body positivity and positive body image are not identical.

As mentioned above, #BodyPostivity aims to encourage adopting an attitude towards the body that will be associated with respect for one’s own body, avoiding constantly making harmful comparisons with others; it encourages having positive beliefs about one’s body and undertaking adaptive behaviors that will be associated with the desire to take care of one’s physical and mental health [1,2,5,6,9]. This social movement is designed to influence various components of the attitude towards the body, including cognitive aspects (body-related beliefs and thoughts—i.e., my body is unattractive/too fat), emotional aspects (body-related feelings and emotions—i.e., shame, fear, blame) and behavioral aspects (body-related actions and behaviors—e.g., physical activity, body grooming) [11]. #BodyPostivity also encourages us to be aware of the commonly existing pressure to change the figure and/or shape of one’s body according to the applicable “standard”, referring mainly to the phenomena of fat shaming or fatphobia (anti-fat bias which is related to prejudicial assumptions concerning people with overweight or obesity, and the mechanism of shaming this group) [12]. Earlier research in this area shows that there is a negative relationship between body appreciation and various dimensions related to attitudes towards the body (e.g., subscales of Sociocultural Attitudes Towards Appearance Questionnaire 4R [13]). However, according to the authors’ knowledge, there are no studies evaluating the relationship between the intensity of body appreciation and variables related to body attitudes in men measured using the Male Body Attitude Scale (especially studies that also refer to beliefs related to the muscularity of the body and the desire to change in this regard).

Another important aspect of the attitude towards one’s own body is muscle dysmorphia. According to DSM V, muscle dysmorphia (MD) is a subtype of body dysmorphic disorder related to the preoccupation with the idea and beliefs that one’s body is not sufficiently muscular and lean [14,15,16,17,18,19,20,21]. It is associated with a strong dependence of one’s self-esteem on physical appearance, with people with MD perceiving their body as small and/or weak, even if their build is average or when they have developed musculature. Therefore, it contributes to taking actions that enable the individual to achieve the desired ideal figure according to their thinking. Importantly, however, the behaviors that people with MD engage in are often compulsive and include, but are not limited to, rigid diet plans, calculation of macro-nutritional values of every item of consumed food, excessive exercise, excessive use of dietary supplements and other substances that may affect the shape of the figure (e.g., anabolic-androgenic steroids). The compulsiveness of the behaviors undertaken is also related to the fact that these people do not give up these activities, even if they see negative consequences for their physical and mental health and their social, sexual, and professional functioning, rest, recreation, etc. People with MD have strong anxiety that their body is not muscular and lean enough, which may be associated with obsessive thoughts about the body and a tendency to avoid situations where their body is exposed to others who may evaluate their appearance [14,15,17,18,19,20,21]. With reference to the above information and the relationship between MD and body appreciation, the only study known to the authors that concerns this relationship is the findings obtained in a group of Lebanese adolescents that indicate a negative relationship between these variables [22]. Studies on adult men seem to be lacking in this context.

In the above information, it is worth noting that the variable associated with the topic of body image is physical activity. Much of the available research, research reviews, and meta-analyses focus on assessing how physical activity is related to a more adaptive body attitude and/or how physical activity interventions can be used to shape a more positive body image [23]. As is well known, motivation for exercise can vary, with many factors influencing this motivation, and one such factor may be our attitude towards our bodies [23,24,25,26,27]. In this context, only a few studies have been conducted so far on the relationship between body appreciation and exercise motives [26,27,28], with these studies showing that there is a positive correlation between body appreciation and functional motives for exercise, for example, health, enjoyment, and challenging oneself [27]. Moreover, a systematic review indicated that there is a positive correlation between the above-mentioned (pro-health and adaptive) types of motivation for exercise and health-oriented exercises and positive body image and healthy eating behaviors, while exercise for reasons related to appearance has a negative correlation with the last two mentioned variables [28]. It is, therefore, worth investigating whether adult men having views in line with those presented in the #BodyPositivity social movement are associated with such motives for exercise that are mentioned in many sources related to #BoPo—that is, pleasure and the desire to take care of the health and functionality of one’s body (often expressed as “nimbleness”).

To sum up, although knowledge about body image, muscle dysmorphia and physical activity is extensive in diverse populations e.g., [16,23,28], research and knowledge on positive body image and muscle dysmorphia, body attitudes and exercise motives in Polish men remain limited. Polish studies published so far have concentrated mainly on the relationship between negative body image and other variables [29,30] or were limited to only women [31] (and as we know, on the one hand, many studies show that women are characterized by a more negative attitude towards their bodies, both in the cognitive, emotional and behavioral aspects, compared to men; on the other hand, these data are often different in relation to the drive for muscularity, e.g., [11,13,17]). Other studies were based on strictly homogeneous samples (e.g., athletes [32]; gym users [33]). In general, there are few studies in the world assessing the importance of the approach to one’s body based on beliefs related to #BodyPositivity for their adaptive functioning in men (including beliefs directly related to muscle dysmorphia, body attitudes and exercise motives). Given the research on women or students regarding the numerous benefits of having a belief-based attitude from the #BodyPositivity [23,24,25,26,27,28], it is vital to develop a fuller understanding of this issue in a sample of adult males. Clarity on this issue is essential to ensure that appropriate interventions are made to improve the functioning of Polish men. When undertaking research in this area, it should also be remembered that many previous analyses indicate that body mass index (BMI)/weight status according to BMI may be important both for body image, muscle dysmorphia and exercise motives and (despite actions related to a #BodyPositivity social movement), people with excessive body weight may still feel stronger pressure to change their physical appearance [16,30,34,35]. Therefore, this work has the following aims: (1) classify different conditions associated with body appreciation and weight status (body mass index; BMI); and (2) analyze and compare the severity of muscle dysmorphia, body attitudes, and exercise motives. We hypothesized (our main hypothesis) that men with high body appreciation and healthy (normal) weight would have significantly lower levels of muscle dysmorphia and the non-adaptive (negative) multiple dimensions of body attitudes, as well as a greater level of the selected pro-health and adaptive exercise motives than men with low body appreciation and excess body weight. Moreover (as part of further analyses), additional comparisons were also made in the above-mentioned areas between the two groups of men already mentioned (men with high body appreciation and healthy weight and men with low body appreciation and excess weight) and the other two groups (men with low body appreciation and healthy weight and men with high body appreciation and excess weight).

## 2. Materials and Methods

### 2.1. Participants and Procedures

Our study was approved by the Ethics Committee (no. 2021/3/4E/5) and all procedures were conducted under the Declaration of Helsinki. The data used in the current analysis were collected from May 2021 to April 2023. Participants were recruited online (e.g., via social media such as Facebook, Instagram) and by on-site flyers (universities and workplace locations). The recruitment strategy was based on using general advertisements and flyers in an academic setting and in general public areas (no inclusion/exclusion criteria beyond BMI considerations—were used). Prior to data collection, all men were informed of the study’s aim and anonymity. Informed consent was sought prior to their participation. (This included assurances that participants could withdraw from the study at any time without incurring any penalties and that the outcomes would only be utilized for research purposes). Next, participants completed our online survey (using Google Forms). Completing the survey took approximately 15–20 min. The participation was voluntary (without remuneration).

Four hundred men volunteered for the study. For the purpose of this analysis, we established a dichotomous variable describing BMI according to the recommendations of the World Health Organization: healthy (normal) vs. excess body weight. Therefore, people with underweight were removed from the database (*N* = 26). Our final sample included 374 Polish men who ranged in age from 18 to 58 years (*M* = 28.96, *SD* = 8.52) and in self-reported body mass index (BMI) from 18.52 to 43.83 kg/m^2^ (*M* = 25.55, *SD* = 3.75). In terms of relationship status, 183 (48.93%) were single, 98 (26.20%) were partnered but not married, 78 (20.86%) were married, and 15 (4.01%) had some other status. In terms of education, 10 (2.67%) had completed primary school, 23 (6.15%) vocational school, 176 (47.06%) secondary school, and 165 (44.12%) had completed an undergraduate degree or completed a Master’s degree.

### 2.2. Measures

#### 2.2.1. The Body Appreciation Scale-2

To measure body appreciation, the participants completed the Body Appreciation Scale-2 (BAS-2) [10]. It consists of 10 items in total, giving a mean score reflecting body appreciation. Participants respond on a 5-point scale (from strongly disagree to strongly agree; example item: I am comfortable in my body). The higher the score, the higher body appreciation. Previous studies indicate that this measure has adequate validity and reliability [10,36]. In our analysis, McDonald’s ω was 0.95.

#### 2.2.2. The Muscle Dysmorphic Disorder Inventory

Muscle dysmorphia was measured using the Muscle Dysmorphic Disorder Inventory (MDDI) [37]. It includes 13 items which, when summed up, give a total score and three subscales: (a) drive for size (example item: I think my chest is too small), (b) appearance intolerance (example item: I wear loose clothing so that people cannot see my body), and (c) functional impairment (example item: I pass up social activities (e.g., watching football games, eating dinner, going to see a movie, etc.) with friends because of my workout schedule). Participants rate statements on a 5-point scale from 1 (never) to 5 (always). The higher the score, the higher the muscle dysmorphia symptoms. Previous studies indicate that this measure has adequate construct and factorial validity and reliability [37,38]. In our analysis, we used total scores and all subscales. The McDonald’s ω values were as follows: ω_total_ = 0.85, ω_drive_ for size = 0.87, ω_appearance_ intolerance = 0.80, and ω_functional impairment_ = 0.85.

#### 2.2.3. The Male Body Attitude Scale

The non-adaptive (negative) multiple dimensions of body attitudes were assessed with the Male Body Attitude Scale (MBAS) [39]. It includes 24 items, providing a total score and three subscales: (a) muscularity (example item: I think I have too little muscle on my body), (b) body fat (example item: I am concerned that my stomach is too flabby), and (c) height (example item: I wish I were taller). Each item is responded to on a 6-point scale from 1 (never) to 5 (always). Mean scores are used with higher scores representing more non-adaptive (negative) body attitudes. Previous studies indicate that this measure has adequate construct and factorial validity and reliability [39]. In our analysis, we used total scores and all subscales. The McDonald’s ω values were as follows: ω_total_ = 0.93, ω_muscularity_ = 0.91, ω_body fat_ = 0.90, and ω_height_ = 0.80.

#### 2.2.4. The Exercise Motivations Inventory-2

To measure exercise motives, participants completed the Exercise Motivations Inventory–2 (EMI-2) [24]. It consists of 51 items, giving 14 subscales (mean score). For each item, a 6-point Likert scale is used, ranging from 0 (not at all true for me) to 5 (very true for me). The higher the score, the stronger the intensity of the selected type of motivation to exercise. In our analysis, we selected three subscales (pro-health and adaptive exercise motives): (a) enjoyment (example item: Because I enjoy the feeling of exerting myself), (b) positive health (example item: Because I want to maintain good health), and (c) nimbleness (example item: To stay/become more agile). Previous studies indicate that this measure has adequate validity and reliability [24,25,40]. The McDonald’s ω values were as follows: ω_enjoyment_ = 0.93, ω_positive health_ = 0.92, and ω_nimbleness_ = 0.91.

#### 2.2.5. The Demographic Survey

Participants also provided the following information: age, relationship status, educational qualification, weight, and height. Weight status (body mass index; BMI) was calculated based on self-reported data. Using the basic recommendations of the World Health Organization [41], participants were divided into two groups: (a) healthy (normal) weight, ≤ 18.50 ≤ BMI ≤ 24.99 kg/m^2^; and (b) excess weight (pre-obesity and obesity), BMI ≥ 25.00 kg/m^2^.

### 2.3. Statistical Analysis

All analyses were conducted using IBM SPSS Statistic version 28. Firstly, to identify clusters (based on body appreciation and weight status), we used a two-step cluster analysis (with Schwarz’s Bayesian criterion). This analysis was chosen as it is appropriate for samples of *N* > 200 and both categorical and continuous variables [42]. Secondly, a multivariate analysis of variance (MANOVA) was used to assess differences between the clusters with regard to muscle dysmorphia, body attitudes and exercise motives. A 5% significance level was used, and (to correct for multiple comparisons) the Bonferroni corrected/adjusted *p*-value was introduced.

## 3. Results

### 3.1. Cluster Analysis of Body Appreciation and Body Mass Index

Four clusters were labeled and characterized as follows: (a) Cluster 1 (*N* = 70), low body appreciation (*M* = 2.59) and healthy (normal) weight (100% participants 18.50 ≤ BMI ≤ 24.99 kg/m^2^); (b) Cluster 2 (*N* = 130), high body appreciation (*M* = 3.99) and excess weight (100% participants BMI ≥ 25.00 kg/m^2^); (c) Cluster 3 (*N* = 58), low body appreciation (*M* = 2.42) and excess weight (100% participants BMI ≥ 25.00 kg/m^2^); and (d) Cluster 4 (*N* = 116), high body appreciation (*M* = 4.25) and healthy weight (100% participants 18.50 ≤ BMI ≤ 24.99 kg/m^2^). Table 1 shows the demographic characteristics of these clusters.

### 3.2. Comparison of the Four Clusters for Muscle Dysmorphia, Body Attitudes and Exercise Motives

MANOVA (using Pillai’s trace) indicated a significant effect of clusters on (a) muscle dysmorphia, (I) V = 0.91, F(3, 368) = 1294.81, *p* < 0.001; (b) non-adaptive (negative) body attitudes, V = 0.94, F(3, 367) = 1453.38, *p* < 0.001; and (c) selected pro-health and adaptive exercise motives, V = 0.86, F(3, 368) = 729.82, *p* < 0.001. Table 2, Table 3 and Table 4 show results with more information about outcomes with Bonferroni’s adjustment for multiple comparisons.

With reference to muscle dysmorphia, our results indicated that Clusters 3 and 4 were significantly different from each other in terms of total score and the subscale for appearance intolerance (lower level of symptoms in the latter Cluster). Cluster 1 and Cluster 2 (with lower levels of symptoms in the latter cluster), as well as Cluster 1 and Cluster 4 (with lower levels of symptoms in the latter cluster) also differed in the same aspects of muscle dysmorphia. In the context of appearance intolerance, Cluster 2 had significantly lower levels than Cluster 3 and higher levels than Cluster 4. In the drive for size subscale, a significant difference was found only between Cluster 1 and 2 (with lower levels of symptoms in the latter cluster). Regarding the other comparisons, no significant differences were found.

With regard to non-adaptive (negative) body attitudes, Cluster 4 had significantly lower levels of total score, body fat and height than Cluster 3. In terms of the same dimensions of negative body attitudes, Cluster 4 also presented significantly lower outcomes than Cluster 1. In terms of total score and the body fat subscale, Cluster 4 also had lower results than Cluster 2, and Cluster 2 was significantly different from Cluster 3 (worse functioning in the second of the mentioned clusters). The last significant difference was between Cluster 1 and Cluster 3 regarding body fat (worse functioning in the second of the mentioned clusters). Regarding the other comparisons, no significant differences were found.

In relation to selected pro-health and adaptive exercise motives, Cluster 4 had higher levels on all subscales than Cluster 3. Similar results were found between Cluster 2 and 3 (worse functioning in the second of the mentioned clusters). In terms of enjoyment and positive health subscales, Cluster 4 also differed significantly from Cluster 1 (worse functioning in the second of the mentioned clusters). The last significant difference was between Cluster 1 and 2 in terms of the enjoyment subscale (worse functioning of the first of the mentioned clusters). With regard to the other comparisons, no significant differences were found.

Overall, our results partially support our hypothesis, as lower levels of some symptoms of muscle dysmorphia and negative body attitudes, as well as higher levels of pro-health and adaptive exercise motives were observed in men with high body appreciation and healthy (normal) weight as compared with men with low body appreciation and excess body weight.

## 4. Discussion

This study aimed to investigate the clusters associated with body appreciation and weight status and compare them in the context of muscle dysmorphia, body attitudes, and exercise motives. The main hypothesis was that men with high body appreciation and healthy (normal) weight would have significantly lower levels of muscle dysmorphia and the non-adaptive (negative) multiple dimensions of body attitudes, as well as greater levels of the selected pro-health and adaptive exercise motives than those of low body appreciation and excess body weight. Four clusters were identified (Cluster 1—low body appreciation and healthy weight; Cluster 2—high body appreciation and excess weight; Cluster 3—low body appreciation and excess weight; Cluster 4—high body appreciation and healthy weight). By analyzing the most important findings, significant differences (in terms of most of the assessed scale/subscales) were found between men who had high body appreciation and healthy weight (Cluster 4) and men with low body appreciation and excess body weight (Cluster 3).

Considering the above-mentioned findings, results consistent with our hypothesis were obtained in terms of muscle dysmorphia on the total score (generally all symptoms expressed by responses to all items) and on one subscale (appearance intolerance). This may mean that men with high body appreciation and healthy weight (compared to those with low body appreciation and excess body weight) were characterized by a lower intensity of the general level of various symptoms of muscle dysmorphia, and (in the context of an appearance intolerance subscale) a lower level of this aspect of muscle dysmorphia, which is related to negative beliefs about body and appearance anxiety or body exposure avoidance. This is expressed by people wearing baggy clothes to the beach and a belief that their body is distasteful and ugly [37].

Outcomes consistent with our hypothesis were not obtained for the other two subscales of the Muscle Dysmorphic Disorder Inventory—drive for size and functional impairment. This might mean that Cluster 4 and Cluster 3 function similarly in these two areas related to muscular dysmorphia. For these subscales, individuals in these clusters would have a similar intensity of thoughts of being smaller/less muscular/weaker, and have similar wishes to increase strength and size. They would have similar desires to smoke and/or have negative emotions in situations that disturb the exercise routine. They would take various harmful actions that are supposed to “protect” against skipping these exercises, even if harmful, for example, to social functioning [18,37]. Interestingly, our results seem to be consistent with recent results in a group of Lebanese adolescents, which show that there is a negative correlation between body appreciation and overall muscle dysmorphic score, and that body appreciation is not significantly related to exercise addiction [22]. Summarizing our outcomes on muscle dysmorphia, one explanation for the findings in this study may be that for men, sharing the views of a #BodyPostivity social movement is protective for men in terms of the general (average) trend associated with this disorder. In particular, this sharing can be important for that aspect of muscle dysmorphia involving fear and body embarrassment, which is related to body fat and avoidance behaviors to hide an “imperfect” physique. This protective effect of posture associated with views overlapping with a #BodyPositivity social movement has not been reported for those aspects of muscle dysmorphia that are related to compulsiveness towards the development of muscle tissue and performing exercise.

Our results partially support our hypothesis also in the context of non-adaptive dimensions of body attitudes because Cluster 4 (compared to Cluster 3) was characterized by lower scores on the general scale and two subscales of the Male Body Attitude Scale—body fat and height. These results (as well as those presented above) indicate that men with high body appreciation and healthy weight (compared to those of low body appreciation and excess body weight) had lower levels of symptoms related to overall negative body attitude and (in relation to the subscales) had lower levels of such aspects of negative body attitudes which are associated with body fat and height (but not muscularity). To the best of the authors’ knowledge, there are no studies analyzing the relationship between body appreciation and the results of the Male Body Attitude Scale. However, other studies (partly relating to similar relationships) show that there is a negative correlation between body appreciation and negative body attitude in both sexes, e.g., [13,43]. Once again, this indicates that views associated with a #BodyPositivity social movement refer to shaping a more adaptive attitude towards the body, but only in relation to general body dissatisfaction and perception of body fat and body weight, but not musculature and potentially harmful behaviors that can be undertaken to develop it.

Importantly, the results obtained in terms of the motives for undertaking exercise confirm the more adaptive and pro-health functioning of Cluster 4 compared to Cluster 3. More specifically, this could mean that exercise by the men in the sample with high body appreciation and health is strongly motivated by enjoyment, a positive attitude towards health, and a desire to be nimble. This is an interesting discovery, because on the one hand, in many places promoting the idea of #BodyPositivity, there is a reference to the fact that physical activity is an important element of taking care of physical and mental health, as well as life comfort; it is suggested that one should look for types of exercises that are pleasant and observe how they change one’s capabilities, e.g., in this context, agility (and not compulsively focus on how body size or weight changes) [5,6,7,9,44,45].

Moreover, the scoping review shows that some studies indicate that a positive body image is associated with greater participation in physical activity and sports [23]. On the other hand, some studies conclude that much of the #BodyPositivity material does not refer to exercise at all. (Cohen et al. [46] indicated that building a positive body image did not include exercise, because this topic appeared only in 4% of the posts in their study.) Moreover, in another study analyzing 141 posts about physical activity with hashtags #BodyPositivity, it was found that half of the posts were about body changes, i.e., the history of how the body shape/weight changed, how hard the person worked for it and what they do to maintain their current appearance [47]. Much of the content analyzed did not seem to be directly related to the above-mentioned movements because the content indicating that a positive attitude towards the body was achieved by disciplining it with strict physical exercises and striving for “standards” of a slim figure. Other posts were related to advertising various products and services (unfortunately, often incompatible with #BodyPositivity). Only five out of all the posts analyzed concerned the enjoyment of doing physical activity itself, rather than doing it only to change the weight and/or shape of the body [47].

Selected additional comparisons between other clusters in terms of muscle dysmorphia and negative body attitudes are reported as follows: (a) Cluster 2 (high body appreciation and excess weight) and Cluster 4 (high body appreciation and healthy weight) achieved similar (not statistically significant) results in five out of eight comparative analyses in the analyzed subscales/scales; (b) Cluster 1 (low body appreciation and healthy weight) and Cluster 4 (high body appreciation and healthy weight) were statistically significantly different in five out of eight statistical analyses (with lower levels of selected aspects of muscle dysmorphia and negative body posture in Cluster 4). Moreover, in terms of pro-health and adaptive exercise motives, we report the following findings: (a) Clusters 2 and 4 did not differ significantly in any of the three comparisons, (b) Cluster 2 and 4 differed in two out of three comparative analyses (higher level of motivation based on enjoyment and positive health in Cluster 4).

Considering the above findings for these additional comparisons between clusters, it can be concluded that the level of body appreciation (i.e., beliefs consistent with a #BodyPostivity) is the key protective factor for the development and/or maintenance of most symptoms related to muscle dysmorphia and a negative attitude towards the body. The level of body appreciation is also the key for building pro-health and adaptive motivation for physical activity, while body weight is less important. However, further analyses should be carried out in this context to look closely at which variables not included in this study could help explain the discrepancies in the results obtained and examine more closely the relationship between the beliefs of the #BodyPostivity movement and the compulsive drive to develop muscle tissue (which seems to be as dangerous as the compulsive pursuit of thinness).

Finally, it should be added that some limitations are present in this study: (1)tThe study was based on self-reported questionnaires and data (note that reliance on self-reported height and weight for BMI calculation may introduce bias leading to underestimation or overestimation of body weight. Note also that BMI does not distinguish body fat from muscularity, which is important to consider when assessing muscle dysmorphia); (2) despite good parameters for validity and reliability in this sample, not all the scales used in this study have been validated in Poland, and when analyzing the results, it must be remembered that a very high value of McDonald’s omega (ω) coefficient might also suggest redundancy in the items (rather than merely acceptable internal consistency); (3) the study is cross-sectional (it is not possible to draw causal relationships; (4) volunteers participated in the study (non-probability sampling); (5) residual confounding bias is possible due to some important mediators/moderators (e.g., body acceptance by others, [27]; eating disorders, [22]), which were not included in the study; (6) the Body Appreciation Scale-2 was not directly designed to assess beliefs related to #BodyPostivity. It should be remembered that body appreciation is only one of the concepts discussed within the body positivity movement and only one of the constructs relating to the assessment of a positive body image. Likewise, body positivity as a social movement and positive body image are not the same, i.e., they are not “substitutes” [48]. Finally, a suggestion for future research may be that in studies similar to ours, other statistical methods such as path analysis to reach a model can be appropriate. Additionally, it may be also worth verifying whether participants were previously exposed to #BodyPositivity content (and if so, to what extent).

## 5. Conclusions

Our results are mostly consistent with the assumed hypothesis because they confirm that (in terms of most of the assessed scale/subscales) men with high body appreciation and healthy (normal) weight have significantly lower levels of muscle dysmorphia and the non-adaptive (negative) multiple dimensions of body attitudes, as well as higher levels of the selected pro-health and adaptive exercise motives, than participants of low body appreciation and excess body weight. These findings show that holding views in line with positive body image is also beneficial for the functioning of adult men, which can be used in interventions and prevention in this group, including activities using social media. An important step may be intervention programs, public health initiatives, or mental health strategies to support men struggling with body image and other concerns in relation to eating disorders, body image dysmorphia, obesity and other mental and physical issues. In this area, the content from #BodyPositivity may be used to create educational materials on men’s body image, with content focusing more on the functionality of our body and the positive components of body attitudes, as well as positive motivations for physical activity (related to feelings of success and enjoyment). However, further research needs to be conducted in this area to determine whether the content in these interventions and prevention activities for both sexes should be the same and what factors should be taken into account so that it is also possible to influence excessive fixation on muscularity.

## Figures and Tables

**Table 1:** Demographic characteristics of the four clusters.

	Cluster 1 (*N* = 70): Low Body Appreciation and Healthy Weight	Cluster 2 (*N* = 130): High Body Appreciation and Excess Weight	Cluster 3 (*N* = 58): Low Body Appreciation and Excess Weight	Cluster 4 (*N* = 116): High Body Appreciation and Healthy Weight	
	*M* (*SD*)				*F*
**Age**	25.94 (6.86) ^aB^	31.06 (9.45) ^aC^	30.48 (8.30) ^B^	27.67 (7.71) ^C^	*F*(3, 370) = 7.43, *p* < 0.001
	*N* (%)				*χ*^2^ and Cramer’s *V*
**Educational qualification**					*χ*^2^_(9)_ = 11.23, Cramer’s *V* = 10 ^NS^
Primary school	4 (5.71)	2 (1.54)	2 (3.45)	2 (1.72)	
Vocational school	3 (4.29)	9 (6.92)	6 (10.34)	5 (4.31)	
Secondary school	38 (54.29)	53 (40.77)	26 (44.83)	59 (50.87)	
Undergraduate degree or completed a Master’s degree	25 (35.71)	66 (50.77)	24 (41.38)	50 (43.10)	
**Relationship status**					*χ*^2^_(15)_ = 30.08, Cramer’s *V* = 0.16 *
Single	43 (61.43)	54 (41.54)	30 (51.73)	56 (48.28)	
Partnered but not married	21 (30.00)	30 (23.08)	12 (20.69)	35 (30.17)	
Married	4 (5.71)	38 (29.23)	15 (25.86)	21 (18.10)	
Other status	2 (2.86)	8 (6.15)	1 (1.72)	4 (3.45)	

^a^—significant differences between the groups with *p* < 0.001; ^B,C^—significant differences between the groups with *p* < 0.01; ^NS^—a non-significant (*p* > 0.05); * *p* < 0.05.

**Table 2:** Separate univariate ANOVAs on muscle dysmorphia.

	Cluster 1 (*N* = 70): Low Body Appreciation and Healthy Weight	Cluster 2 (*N* =130): High Body Appreciation and Excess Weight	Cluster 3 (*N* = 58): Low Body Appreciation and Excess Weight	Cluster 4 (*N* = 116): High Body Appreciation and Healthy Weight	
	*M* (*SD*)				*Post hoc*
**Muscle Dysmorphic Disorder Inventory**					
	*F*(3, 370) = 6.71, *p* < 0.001, *ƞ_p_*^2^ = 0.05				
Total score	34.66 (9.92)	30.70 (9.47)	34.38 (10.06)	29.08 (10.04)	1 vs. 2 * 1 vs. 3 1 vs. 4 ** 2 vs. 3 2 vs. 4 3 vs. 4 **
	*F*(3, 370) = 2.71, *p* < 0.05, *ƞ_p_*^2^ = 0.02				
Subscale: drive for size	14.67 (4.85)	12.54 (5.00)	12.78 (5.37)	13.13 (5.48)	1 vs. 2 * 1 vs. 3 1 vs. 4 2 vs. 3 2 vs. 4 3 vs. 4
	*F*(3, 370) = 37.00, *p* < 0.001, *ƞ_p_*^2^ = 0.23				
Subscale: appearance intolerance	10.47 (3.49)	8.49 (3.06)	11.97 (3.55)	6.96 (3.17)	1 vs. 2 *** 1 vs. 3 1 vs. 4 *** 2 vs. 3 *** 2 vs. 4 ** 3 vs. 4 ***
	*F*(3, 370) = 0.63, *p* > 0.05, *ƞ_p_*^2^ = 0.001				
Subscale: functional impairment	9.51 (4.15)	9.67 (3.95)	9.64 (3.79)	8.99 (4.56)	1 vs. 2 1 vs. 3 1 vs. 4 2 vs. 3 2 vs. 4 3 vs. 4

* *p* < 0.05, ** *p* < 0.01, *** *p* < 0.001.

**Table 3:** Separate univariate ANOVAs on non-adaptive (negative) body attitudes.

	Cluster 1 (*N* = 70): Low Body Appreciation and Healthy Weight	Cluster 2 (*N* =130): High Body Appreciation and Excess Weight	Cluster 3 (*N* = 58): Low Body Appreciation and Excess Weight	Cluster 4 (*N* = 116): High Body Appreciation and Healthy Weight	
	*M* (*SD*)				*Post hoc*
**Male Body Attitude Scale**					
	*F*(3, 370) = 18.18, *p* < 0.001, *ƞ_p_*^2^ = 0.13				
Total score	3.42 (1.03)	3.09 (0.94)	3.80 (1.06)	2.73 (0.90)	1 vs. 2 1 vs. 3 1 vs. 4 *** 2 vs. 3 *** 2 vs. 4 * 3 vs. 4 ***
	*F*(3, 370) = 3.20, *p* < 0.05, *ƞ_p_*^2^ = 0.03				
Subscale: muscularity	3.59 (1.29)	3.14 (1.19)	3.51 (1.18)	3.15 (1.21)	1 vs. 2 1 vs. 3 1 vs. 4 2 vs. 3 2 vs. 4 3 vs. 4
	*F*(3, 370) = 29.97, *p* < 0.001, *ƞ_p_*^2^ = 0.20				
Subscale: body fat	3.18 (1.19)	3.26 (1.13)	4.14 (1.28)	2.45 (1.00)	1 vs. 2 1 vs. 3 *** 1 vs. 4 *** 2 vs. 3 *** 2 vs. 4 *** 3 vs. 4 ***
	*F*(3, 370) = 9.27, *p* < 0.001, *ƞ_p_*^2^ = 0.07				
Subscale: height	2.71 (1.11)	2.39 (0.90)	2.74 (0.92)	2.09 (0.87)	1 vs. 2 1 vs. 3 1 vs. 4 *** 2 vs. 3 2 vs. 4 3 vs. 4 ***

* *p* < 0.05, *** *p* < 0.001.

**Table 4:** Separate univariate ANOVAs on selected pro-health and adaptive exercise motives.

	Cluster 1 (*N* = 70): Low Body Appreciation and Healthy Weight	Cluster 2 (*N* =130): High Body Appreciation and Excess Weight	Cluster 3 (*N* = 58): Low Body Appreciation and Excess Weight	Cluster 4 (*N* = 116): High Body Appreciation and Healthy Weight	
	*M* (*SD*)				*Post hoc*
**Exercise Motivations Inventory-2**					
	*F*(3, 370) = 11.09, *p* < 0.001, *ƞ_p_*^2^ = 0.08				
Subscale: enjoyment	2.52 (1.60)	3.42 (1.44)	2.37 (1.49)	3.33 (1.46)	1 vs. 2 *** 1 vs. 3 1 vs. 4 ** 2 vs. 3 *** 2 vs. 4 3 vs. 4 ***
	*F*(3, 370) = 6.58, *p* < 0.001, *ƞ_p_*^2^ = 0.05				
Subscale: positive health	3.28 (1.58)	3.77 (1.33)	3.03 (1.38)	3.86 (1.33)	1 vs. 2 1 vs. 3 1 vs. 4 * 2 vs. 3 ** 2 vs. 4 3 vs. 4 **
	*F*(3, 370) = 5.31, *p* < 0.01, *ƞ_p_*^2^ = 0.04				
Subscale: nimbleness	2.71 (1.56)	3.16 (1.54)	2.24 (1.38)	2.97 (1.52)	1 vs. 2 1 vs. 3 1 vs. 4 2 vs. 3 ** 2 vs. 4 3 vs. 4 *

* *p* < 0.05, ** *p* < 0.01, *** *p* < 0.001.

## Data Availability

The datasets generated and/or analyzed during the current study are not publicly available due to ethical issues involving participants’ data and privacy but are available from the corresponding author on reasonable request.

## Abbreviations

The following abbreviations are used in this manuscript:

BMI	index
M	mean
SD	standard deviation
MD	muscle dysmorphia
N	total number of individuals
V, F	statistics for MANOVA (using Pillai’s trace)
P	*p*-value
vs.	versus
ƞ_p_^2^	effect size
BAS-2	Body Appreciation Scale-2
MDDI	Muscle Dysmorphic Disorder Inventory
MBAS	Male Body Attitude Scale
EMI-2	Exercise Motivations Inventory–2
MANOVA	multivariate analysis of variance
